# A Remote Lab for Experiments with a Team of Mobile Robots

**DOI:** 10.3390/s140916486

**Published:** 2014-09-04

**Authors:** Marco Casini, Andrea Garulli, Antonio Giannitrapani, Antonio Vicino

**Affiliations:** Dipartimento di Ingegneria dell'Informazione e Scienze Matematiche, Università di Siena, Via Roma 56, Siena 53100, Italy; E-Mails: garulli@ing.unisi.it (A.Ga.); giannitrapani@ing.unisi.it (A.Gi.); vicino@ing.unisi.it (A.V.)

**Keywords:** remote labs, control education, multi-robot systems, mobile robotics

## Abstract

In this paper, a remote lab for experimenting with a team of mobile robots is presented. Robots are built with the LEGO Mindstorms technology and user-defined control laws can be directly coded in the Matlab programming language and validated on the real system. The lab is versatile enough to be used for both teaching and research purposes. Students can easily go through a number of predefined mobile robotics experiences without having to worry about robot hardware or low-level programming languages. More advanced experiments can also be carried out by uploading custom controllers. The capability to have full control of the vehicles, together with the possibility to define arbitrarily complex environments through the definition of virtual obstacles, makes the proposed facility well suited to quickly test and compare different control laws in a real-world scenario. Moreover, the user can simulate the presence of different types of exteroceptive sensors on board of the robots or a specific communication architecture among the agents, so that decentralized control strategies and motion coordination algorithms can be easily implemented and tested. A number of possible applications and real experiments are presented in order to illustrate the main features of the proposed mobile robotics remote lab.

## 1. Introduction

Recent years have witnessed an impressive flourishing of remote laboratories for teaching and research purposes (see, e.g., [1–4]). Remote labs allow students and researchers to address engineering problems with real-world experiments, which are made available through the Internet by physical laboratories spread all over the world. Students have the opportunity to practice subjects taught in classes without worrying about the experimental setup, while researchers can test new ideas on testbeds which have been already used by other research groups, thus producing quick and reliable comparisons of different solutions. In many cases, remote labs have proven to be a successful trade-off between the need for practical experiences and the shortage of resources or time.

A large number of remote labs are available nowadays in the fields of control and robotics, where experimental validation plays a key role. This is the case, for example, for control applications, in which accurate plant models are not available or difficult to estimate, and it is therefore customary to bridge the gap between simulations and experimental tests on the physical process (see, e.g., [5–9] and references therein).

Given the importance of experimentation in robotics, a number of virtual labs and remote experimental setups have been designed for education in this field (see, e.g., the special issue [10] and references therein). In [11] the educational impact of real, virtual and remote labs on the teaching of robotics disciplines is compared. A remote lab devoted to programming embedded real-time systems is described in [12], where teaching experiences with students programming a robotic arm to execute coordinated tasks are reported. Another remote lab for interacting with a real robot has been proposed in [13]. In this case, a PR2 robot (a two-armed robot with an omnidirectional base) is made available for experimentation. An interesting feature in the implementation of this lab is the adoption of the Robot Operating System (ROS) [14] as an open source middleware infrastructure to enhance flexibility and interoperability. In the same spirit, is the recent work [15], where an open source architecture for easily deploying remote robotics labs is presented. The proposed web-based framework can accommodate any robotic device running the ROS operating system and, as such, is suited to perform a variety of robotics experiments. As an example, this infrastructure has been exploited to construct a 3D object recognition database for grasping purposes by means of crowdsourcing, *i.e.*, by obtaining information from a large number of people through the Internet [16].

When focusing on mobile robotics, only few specific remote labs are available. In this domain, experimental setups are usually expensive and difficult to maintain due to space and technological requirements. An example in this respect is the framework for tele-experiment in mobile robotics proposed in [17], which takes explicitly into account the limitations introduced by the communication architecture and constraints. The SyRoTek e-learning platform described in [18], which features a team of small mobile robots performing navigation tasks in a restricted arena with obstacles. In [19], the authors present a remote lab in which a tele-operated real robot has to construct a map of the environment by exploiting noisy sensor measurements.

This paper presents a remote lab for mobile robotics, which enjoys all the benefits of practicing with real robots, while at the same time minimizing the amount of work required to build a fully functional setup for multi-agent systems. The mobile robots are based on the Lego Mindstorms NXT technology, which is becoming a widespread standard in control and robotics education (see, e.g., [20,21]). The remote lab exploits the Matlab environment for the design of control strategies for the multi-robot team. Users access the lab through standard web browsers, and a graphical interface allows them to select one out of a set of predefined experiments, or to test user-defined controllers by uploading a Matlab function. During the experiments, real-time visual feedback is ensured by video streaming. Relevant data are depicted on-line in a dedicated window and can be downloaded at the end of the experiment for offline analysis. An automatic battery recharge procedure (exploiting a suitable recharge station built on purpose) is provided, to guarantee long term availability of the experimental testbed. The position and orientation of each robot is available to the user in real time, so that it is possible to simulate the presence of different types of exteroceptive sensors (e.g., sonars, range finders, *etc.*) on board of the robots. Similarly, the user can simulate a specific communication architecture allowing the agents to exchange information about their state, including features such as point-to-point communication links between robot pairs, communication failures, noisy channels and so on. This allows the user to design and test a number of decentralized multi-agent control techniques or motion coordination algorithms, in the presence of a realistic sensorial and communication infrastructure. The proposed multi-robot setup is embedded in the Automatic Control Telelab (ACT) [5,6], a remote laboratory developed at University of Siena for teaching purposes, aimed at providing a variety of remote experiments in the control systems field. The addition of such a facility enlarges the remote lab audience, attracting also students involved in robotics courses. Thanks to its architecture, the proposed setup can be effectively used for both educational purposes (e.g., remote exercises) and research activities (e.g., experimental testing of innovative control laws). Besides presenting the main features of the multi-robot setup, a further objective of the paper is to illustrate several possible experiences available to the user, such as multi-agent coordination and decentralized control, pursuer-evader games and collision avoidance problems, and to show how they can be useful both for research and for teaching purposes.

The paper is organized as follows. Section 2 introduces the multi-robot remote lab facility, describing both hardware and software architectures. A typical interactive session is presented in Section 3. Several applications available in the multi-robot remote lab are outlined in Section 4, while some related experiments and teaching experiences are summarized in Section 5. Concluding remarks and possible future developments are reported in Section 6.

## 2. System Overview

The main target of the remote lab is to provide an easy-to-use and powerful tool for performing multi-robot experiments through the web. In the mobile robotics field, it is well-known that testing control algorithms on real devices is in general a time consuming and costly task. By using the proposed facility, one may perform a number of different experiments with a minimal effort, thus saving time and money. Users may connect to the lab, upload a Matlab function implementing the desired multi-robot control strategy, run the remote experiment, watch its behavior, and finally download the recorded data.

**Figure 1.**
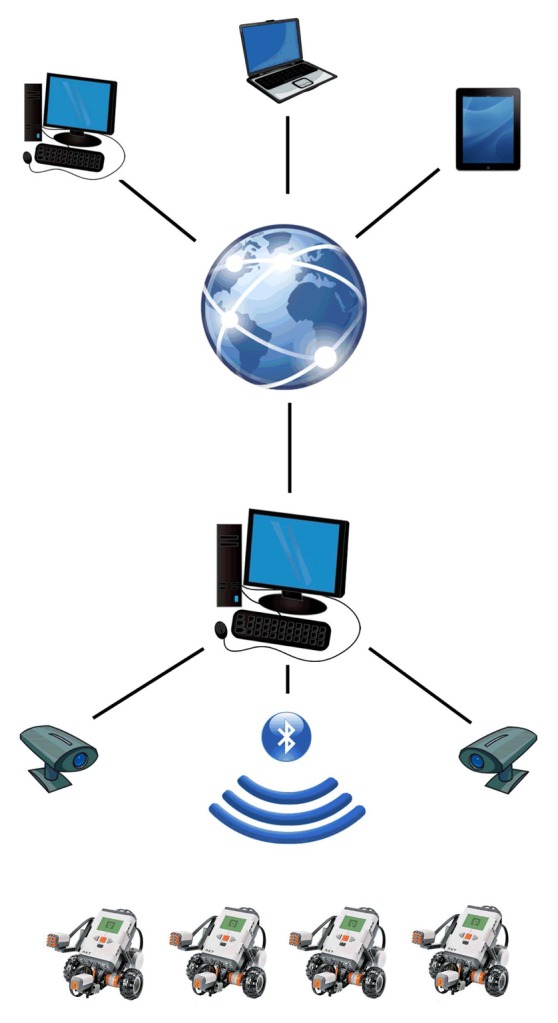
Hardware architecture.

In Figure 1, a sketch of the developed setup is depicted. In the following, a detailed description of the hardware and software architecture is reported.

### 2.1. Hardware Description

**Figure 2.**
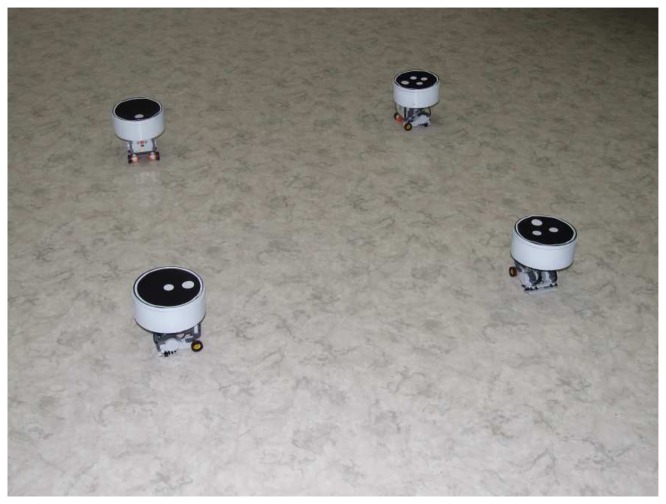
The LEGO NXT mobile robots.

The proposed setup consists of four identical mobile robots able to move in a workspace of 4.5 × 3 m. Robots are built by using the LEGO Mindstorms technology. They are equipped with two motors able to drive the left and right wheels, and a steel ball transfer unit acts as third support. The core of each vehicle is the NXT brick, which hosts a microprocessor providing computation capabilities, motor actuation, and Bluetooth communication. Special markers are placed on the top of each vehicle (see Figure 2) allowing for the visual detection of the robot poses. Robots have been designed to prevent overturn in case of collision. Mechanical characteristics of vehicles are reported in Table 1.

**Figure 3.**
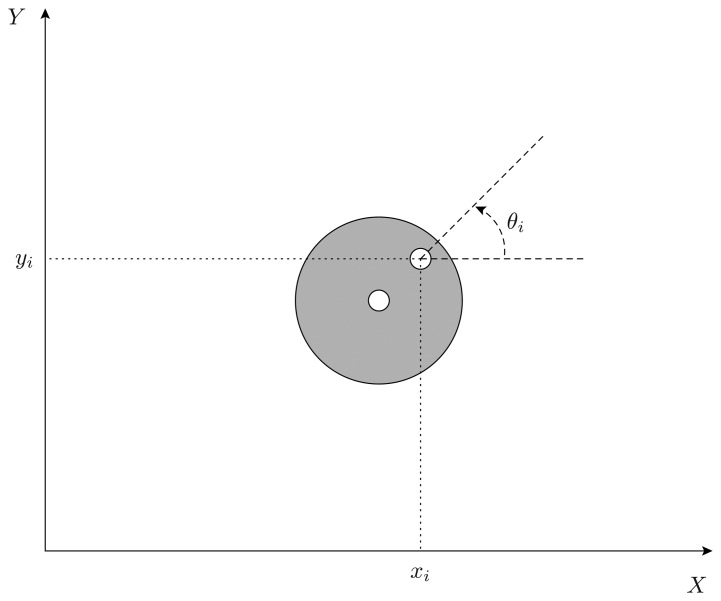
Sketch of robot pose. The shape of the robot reflects the top view of a real vehicle.

The four vehicles feature a unicycle-like kinematics which is quite common in practice. Denoting by *p_i_*(*t*) = [*x_i_*(*t*) *y_i_*(*t*) *θ_i_*(*t*)]′ the position and orientation of the *i*-th robot at time *t* (see Figure 3), the robot pose evolves according to the model

(1) $$\begin{array}{ll} \begin{array}{l} {\dot{x}}_{i}(t)=\upsilon_{i}(t)\cos(\theta_{i}(t)),\\ {\dot{y}}_{i}(t)=\upsilon_{i}(t)\sin(\theta_{i}(t)),\\ {\dot{\theta }}_{i}(t)=\omega_{i}(t), \end{array} & i=1,\ldots ,4 \end{array}$$

where *υ*_i_ and *ω_i_* are the linear and angular speed, respectively.

Low-level control of the right and left wheel of each vehicle is in charge of a program running on the NXT, written in the NXC (Not eXactly C) language. Such a program implements a PI-controller able to make the wheel speed track a reference signal received through the Bluetooth connection.

In order to detect robot position and orientation, a visual system composed of two wide-angle cameras placed on the lab ceiling is employed. Using two cameras allows one to enlarge the field at the price of a higher computational effort.

To ensure long term availability of the system, a crucial aspect regards automatic recharging of robot batteries. To this purpose, once an experiment is over, an *ad*-*hoc* procedure drives the robots to a “box” able to recharge their lithium batteries without any human intervention, thanks to two metal plates placed on the bottom of each robot.

### 2.2. Software Description

Nowadays, many software technologies are available for the development of remote labs (see, e.g., [22,23]). The solution adopted here consists in using a client-server architecture, where the server is implemented as a Matlab function performing the following tasks.

1. *Robot detection.* To detect the robot poses, the images of the two wide-angle cameras are captured by using the Matlab *Image Acquisition Toolbox* and they are filtered to extract the markers of each robot by using functions for feature extraction provided by the Matlab *Image Processing Toolbox*. An offline calibration procedure was performed to estimate the camera parameters in order to correct the acquired images affected by barrel distortion. At this point, the two corrected images are merged and the global coordinates are computed through suitable homogeneous transformations. Moreover, a compressed version of these images is stored and used for online video streaming.
2. *Robot control.* User-defined controllers must be implemented as a Matlab function which will be executed at each time step. Based on the state of the multi-agent system, this function has to return the desired linear and angular velocities for each robot. Through this solution, users develop their own control law by taking advantage of the wide set of powerful functions already provided in Matlab and its toolboxes. Moreover, being Matlab a standard tool in the control systems and robotics community, such a choice is convenient from the educational point of view, allowing students to reduce the time needed to implement their own multi-robot control strategy.
3. *Communication.* Communication to robots is performed through the Bluetooth protocol. At each sampling time, the desired robot speed computed by the user-defined controller is sent to each vehicle. Moreover, robots can send messages to the server to inform it about their state, e.g., their battery level.
4. *TCP connection.* Interaction with the user is achieved through a TCP connection between the running Matlab function and a Java applet providing a graphical user interface (GUI, see Section 3). The experiment can be started or stopped by the user, while during an experiment, vehicle poses are sent by the server to the Java applet for online visualization.
5. *Data storage.* During an experiment, all the relevant data such as robot poses and computed commands are stored in a Matlab structure. When the experiment is over, users may download such data to perform offline analysis.
6. *Automatic battery recharge.* When an experiment is over, robots are driven to the recharge station. Correct recharge is checked periodically by reading the battery level of each robot.
7. *Safety system.* To prevent robots from getting lost, the experiment is stopped whenever a robot is going outside the workspace or when any error is detected (e.g., TCP communication drops). In these cases, the experiment is stopped and a procedure is launched which drives the robots to a safe position.

Besides the previously described Matlab functions, a standard web server with PHP capabilities (Apache web server) is running on the remote lab server. It allows users to connect to the remote lab, to browse the site, to upload the controller and to download the experiment data.

## 3. Session Description

The only requirement needed by the user in order to perform remote experiments on the team of robots is a web browser with Java capabilities. If a user wants to test a custom controller via simulations before submitting it to the remote lab server, the Matlab environment is required. From the main page of the mobile robotics section of the remote lab, users may choose the type of experiment to perform, like generic experiments in empty environment or collision avoidance experiments with virtual obstacles.

**Figure 4.**
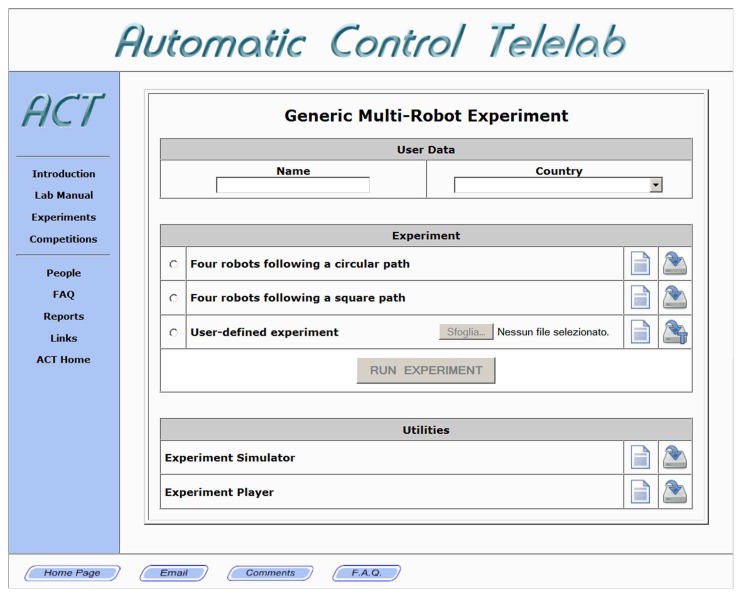
The Control Interface.

**Figure 5.**
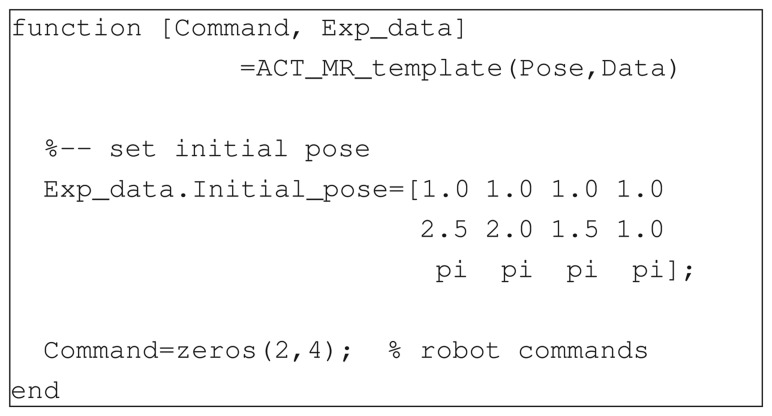
Template function for user-defined controllers.

When selecting the generic multi-robot experiment, the user is presented with the *Control Interface* (Figure 4). The user can choose the controller to be used in the experiment among a set of predefined control laws or a custom one. Predefined experiments are provided both for showing the capabilities of the proposed remote lab and for helping users design their own tests. To perform a user-defined experiment, one has to download and modify the provided template (Figure 5). The syntax of this Matlab function is very simple. Input parameters are the robot poses (defined by the 3×N_robot matrix Pose whose *i*-th column is *c_i_* = [*x_i_*(*t*), *y_i_*(*t*), *θ_i_*(*t*)]′, N_robot being the number of robots involved in the experiment) and a data structure (Data) containing information about the state of the experiment (e.g., current time, sampling time, *etc.*). Output arguments are the desired linear and angular speed of each robot, embedded in the 2×N_robot matrix Command, and an optional structure containing other information like the robot initial conditions. This type of user interaction gives the system a high level of versatility, allowing users to practice with a number of different problems related to multi-agent systems, ranging from single-robot to multi-robot experiences, from centralized to decentralized control experiments.

**Figure 6.**
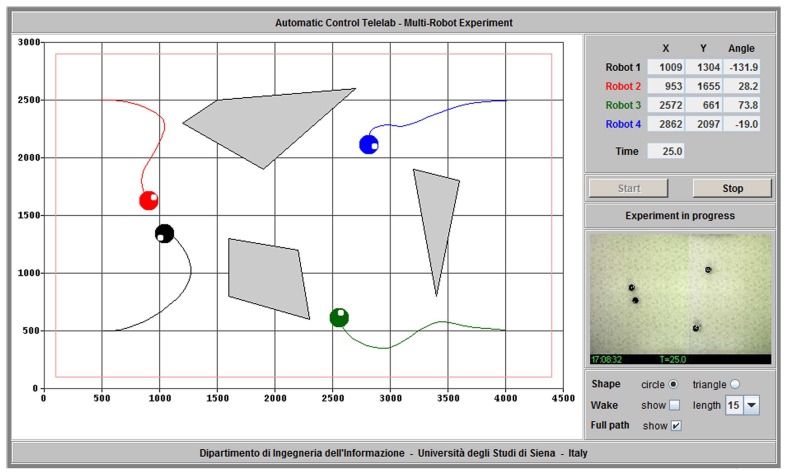
The user interface.

Once a control law has been selected, a GUI shows up (Figure 6). This interface has been developed as a Java applet to guarantee platform independence. From this GUI, a user may start and stop the experiment, watch a graphical representation of the experiment and a streaming video.

**Figure 7.**
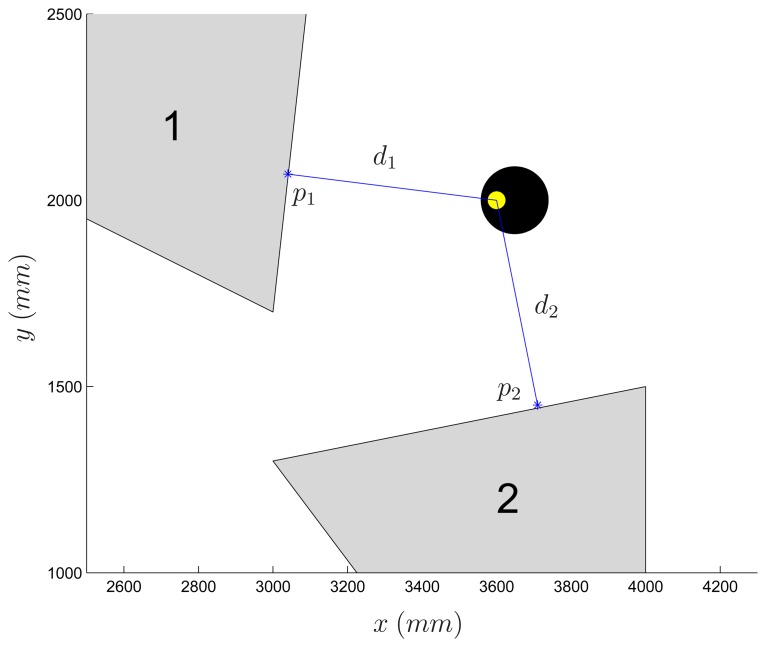
Virtual obstacles: for each robot, the minimum distance to each obstacle (*d_i_*, *i* = 1,2) and the point of minimum distance (*p_i_*, *i* = 1,2) are computed and stored into a suitable Matlab structure.

The experiment reported in Figure 6 is related to a path planning task in the presence of *virtual obstacles*. In order to test more advanced algorithms involving a non empty workspace, users may define an arbitrary number of polygonal obstacles or pick up one out of a set of predefined environments. During the experiment, at each time step the Robot Detection module computes additional information related to the virtual obstacles which are then made available to the Robot Control function. Specifically, for each robot, the distance to each obstacle and the obstacle point corresponding to the minimum distance to the robot are computed (see Figure 7). The inter-vehicle distance is computed as well, thus allowing to treat robots as moving obstacles and easing the implementation of algorithms preventing robot collisions. Finally, also a constantly updated occupancy grid is provided to the Robot Control function. The whole workspace is partitioned into equally sized cells, each of them being marked with a binary value which indicates whether it is currently free or occupied by a virtual obstacle or a robot.

When the experiment is over, a file in Matlab workspace format (.mat) containing all the relevant data of the experiment can be downloaded for offline analysis.

In addition to the remote facilities described, two additional tools can be downloaded and used locally, to help users in designing and evaluating their algorithms, namely the *System Simulator* and the *Experiment Player*. Each of them consists in a Matlab function.

The System Simulator takes as input the designed control function and simulates the behavior of the system returning a graphical animation of robots as well as numerical data. This tool is extremely useful before testing a user-defined controller on the real setup, in order to guarantee the formal correctness of the function (e.g., avoiding syntax errors) and to show the predicted robot trajectories. It is worthwhile to note that, being a simulation, it is not guaranteed that the real experiment follows exactly the predicted one (due to noise, model inaccuracies, *etc.*), but in any case it gives a flavor about the behavior of the multi-robot system.

Through the Experiment Player it is possible to graphically playback the real experiment behavior from the downloaded data. Moreover, one may use it also for creating an animation of the experiment. All the experiment representations reported in Section 5 have been generated by using this tool.

## 4. Possible Applications

Thanks to its high versatility, the proposed setup can be used to carry out a variety of experiments. In the following, three classes of possible applications are outlined.

### 4.1. Pursuer-Evader Games

In many problems involving multi-agent systems, the agents compete with each other to pursue their own objectives (non-cooperative scenario). A typical example is the pursuer-evader (PE) problem. It was first introduced in the context of differential game theory with the aim of determining necessary conditions for two moving agents to collide [24]. Since then, a number of different versions of the problem have been addressed. When dealing with mobile robots, the motion domain is naturally continuous and several additional aspects must be taken into account, such as the kinematic constraints which often limit the maneuverability of real vehicles [25], or the presence of sensors featuring limited field of view [26].

In order to exploit the developed experimental setup for designing and testing PE strategies, it is worth noting that the robots feature the unicycle-like kinematics Equation (1). This motion model is quite common in practice and suitable, for example, for the control laws proposed in [25,27]. Conversely, the boundedness of the available free space poses some limitations on the feasible PE strategies. For example, in this case it seems reasonable to assume that the evader is faster than the pursuer, which is the opposite of what is usually done when moving in free space like in [25]. This is a further challenge the user has to face when playing the role of the pursuer in the proposed setup.

The competitive nature of the PE problem makes it suitable to several teaching experiences where students have to play against computer-controlled opponents or between each other.

### 4.2. Collision Avoidance

The collision avoidance problem, which represents one of the most basic issues in autonomous navigation, is another good example of application of the remote lab for teaching purposes. An essential condition for the successful operation of robots moving in non-empty environments is the safety requirement, *i.e.*, the ability of avoiding collisions which could cause damages to things or people. Several collision avoidance algorithms have been investigated since long time, for different kinds of robots and environments (see [28] for a comprehensive treatment of robot motion planning techniques). One of the first and most intuitive obstacle avoidance algorithms is based on the concept of *artificial potential field*, originally proposed in [29]. In this framework, the robot is assimilated to a particle immersed in a potential field, and, as such, it moves according to a force which is the negative gradient of the potential function. By suitably shaping the artificial potential it is possible to drive the robot towards the target, while at the same time avoiding collisions with the obstacles. An alternative approach consists of using extension theory in conjunction with ultrasonic sensor measurements [30]. When dealing with a team of robots, these techniques can be suitably adapted to safely achieve desired geometrical configurations. Collision avoidance strategies for one or more robots can be easily tested in the remote lab. Users can conveniently define a map of the environment in terms of virtual obstacles, and then exploit the information that the system automatically makes available, like the distance between robots and obstacles, to implement their favorite collision avoidance algorithms.

### 4.3. Multi-Agent Motion Coordination

**Figure 8.**
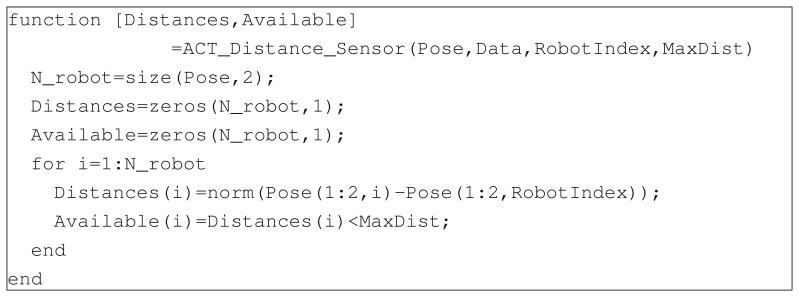
Example of function for distance sensor.

Research-oriented applications of multi-agent systems like environmental monitoring, remote sensing or exploration missions, require the motion of the team to be properly controlled to optimize the performance (e.g., minimize completion time or maximize coverage), while at the same time guaranteeing safety (e.g., avoid collisions among agents). The proposed setup allows one to test both centralized and decentralized control architectures. In the former case, one assumes the existence of a central unit having access to the state of the whole team. Given such information, the control inputs of each agent are computed and sent back to the robots. While such an approach favors simplicity and safety, it has the main drawback to be not tolerant to failures of the central unit or of the communication infrastructure. For this reason, attention of the researchers has focused on the development of decentralized control laws, in which each agent computes its inputs on the basis of its own state and that of a limited number of neighbors. In this scenario, the motion coordination problem is much harder and current research focuses on developing suitable controllers featuring desirable properties like stability of the formation or optimality of the trajectories (see, e.g., [31–33]). Notice that decentralized control laws are based on the information either gathered by the on-board sensors or exchanged among agents. Although the robots do not carry any of such exteroceptive sensors, and cannot directly communicate among themselves, the experimental setup is flexible enough to allow one to simulate the presence of *virtual sensors* or inter-vehicle communication, along with the corresponding technological limitations. In fact, from the pose of the vehicles provided by the vision system, the required measurements can be easily generated via software, thus allowing the user to simulate various types of on-board sensors (e.g., laser rangefinders, sonars, *etc.*) with different levels of sensor accuracy. Then, the measurements can be processed according to the preferred sensor fusion approach, taking into account which ones are available to each agent. The control law of each robot is computed on the basis of the locally available information, thus preserving the decentralization of the algorithm. As an example, Figure 8 reports the code for simulating a sensor detecting the distance from one robot to the others, if the relative distance is less then a prescribed tolerance MaxDist.

## 5. Experiments

In this section, the results of some real experiments are reported in order to illustrate the wide spectrum of experiences that can be proposed through the considered facility. In particular, we will first illustrate a pursuer-evader problem, which can be proposed to both high-school and graduate students. Then, obstacle avoidance techniques can be designed and tested, thanks to the possibility of including virtual obstacles in the environment. Finally, some multi-agent motion coordination experiments will be illustrated. Other examples can be found in [34].

### 5.1. Pursuer-Evader Games

The pursuer-evader game is a framework which allows one to create several educational scenarios suited for students ranging from high-school to graduate and post-graduate. In fact, depending on the given experiment and the chosen role, one can devise several tasks with different complexity. Moreover, for a given task, the difficulty can be adjusted by varying the speed ratio between players. In the following, two examples with increasing complexity are reported to show the potential of the proposed facility as a tool for control education on mobile robotics.

Some experiments concerning PE games were conducted by a team of high school students, within a teaching program aimed at stimulating students' interest towards scientific and technological university curricula.

**Figure 9.**
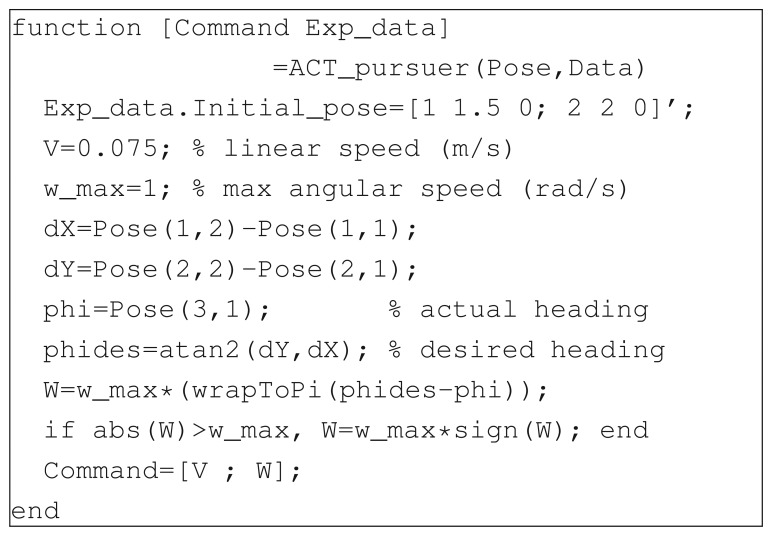
Pursuer-evader games: the Matlab function implementing the pursuer algorithm.

In this respect, the use of a web-based remote lab to perform real experiments, and in general the adoption of tools borrowed from the IT field, is an effective way to attract students' attention and make scientific studies more appealing (see, e.g., [35]). It was asked to three groups of students to design an evader algorithm able to survive a pursuer within an empty environment. The pursuer strategy, unknown to the students, was designed to make the pursuer continuously point towards the evader at maximum speed (see Figure 9 for the Matlab code). To ease the task, a predefined function able to move the robot to a desired pose was provided to the students. This way, the control function only had to return a desired reference pose for the evader, rather then the actual linear and angular velocity.

**Figure 10.**
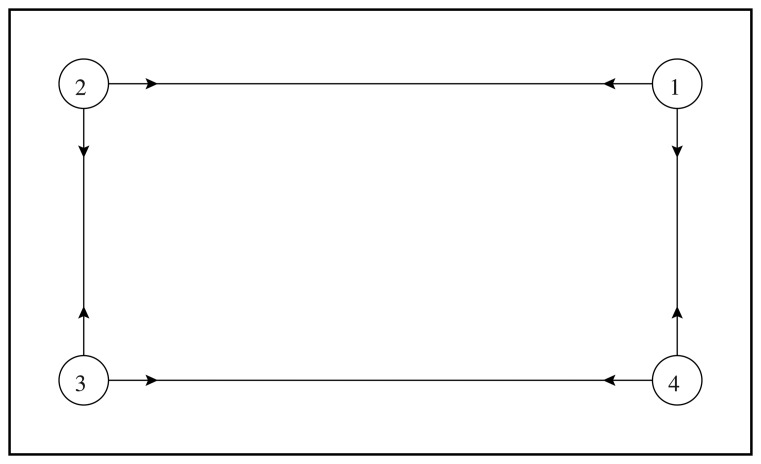
Pursuer-evader games: the evader strategy implemented in Algorithm 1.

**Figure 11.**
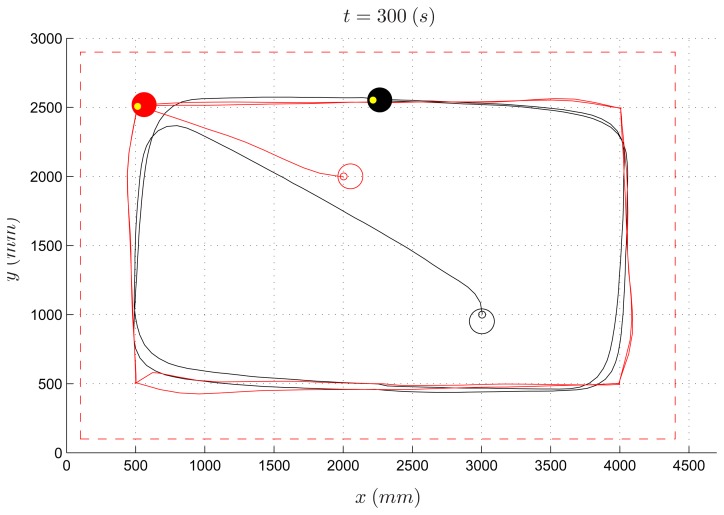
Pursuer-evader games, Algorithm 1: pursuer (black) and evader (red) with a PE speed ratio equal to 50%.

**Figure 12.**
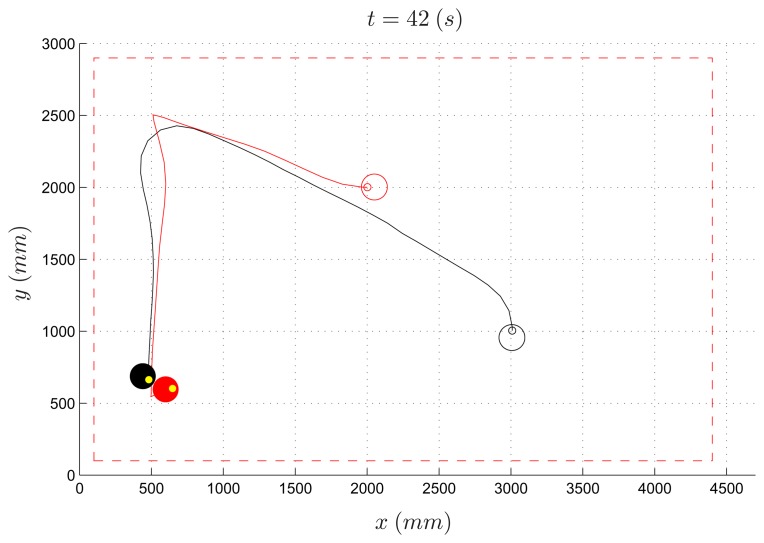
Pursuer-evader games, Algorithm 1: pursuer (black) and evader (red) with a PE speed ratio equal to 80%.

Among the algorithms designed by the three groups of students, the one which performed best (hereafter named *Algorithm 1*) forces the evader to move to one out of four places near the vertexes of the workspace, depending on the current position of the pursuer (Figure 10). The length of the experiment was set to 300 s. Despite the simplicity of the proposed strategy, this algorithm turns out to work well, and it was able to let the evader survive when the speed of the pursuer is less than 70% of the evader speed. In Figures 11 and 12, the path of the two vehicles are reported for a PE speed ratio of 50% (evader wins) and 80% (pursuer wins), respectively.

**Figure 13.**
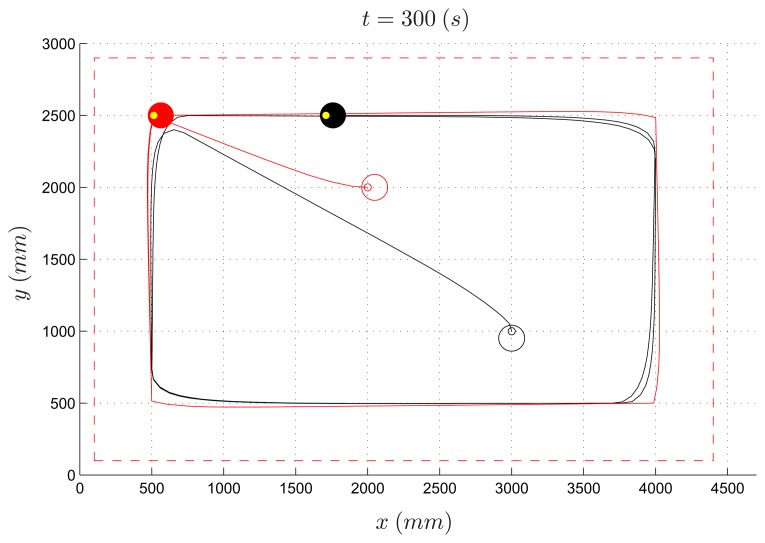
Pursuer-evader games, Algorithm 1: pursuer (black) and evader (red) with a PE speed ratio equal to 50% (simulated experiment).

From an educational viewpoint, it is interesting to compare the real experiments to the results obtained through the provided simulator. In Figure 13, the simulated path of the two vehicles are reported for a PE speed ratio of 50%. Notice that, for this experiment, robot trajectories in Figures 11 and 13 are similar and the simulated experiment provides a good approximation of reality.

**Figure 14.**
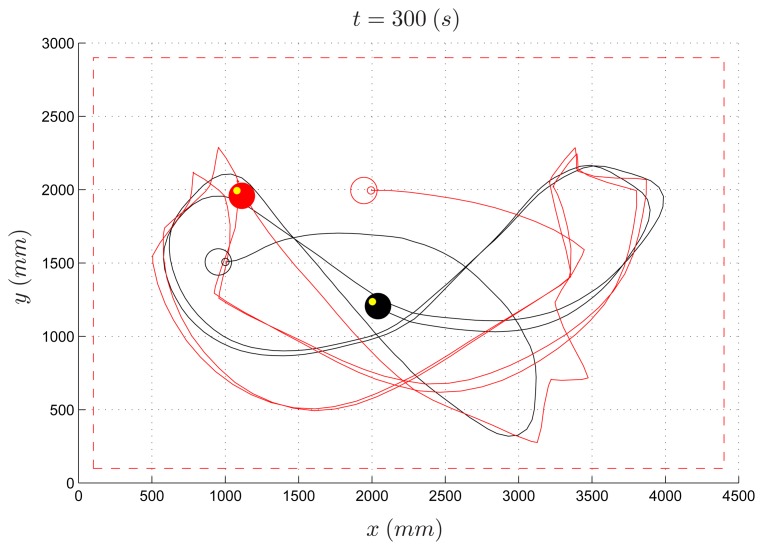
Pursuer-evader games, Algorithm 2: pursuer (black) and evader (red) with a PE speed ratio equal to 50%.

**Figure 15.**
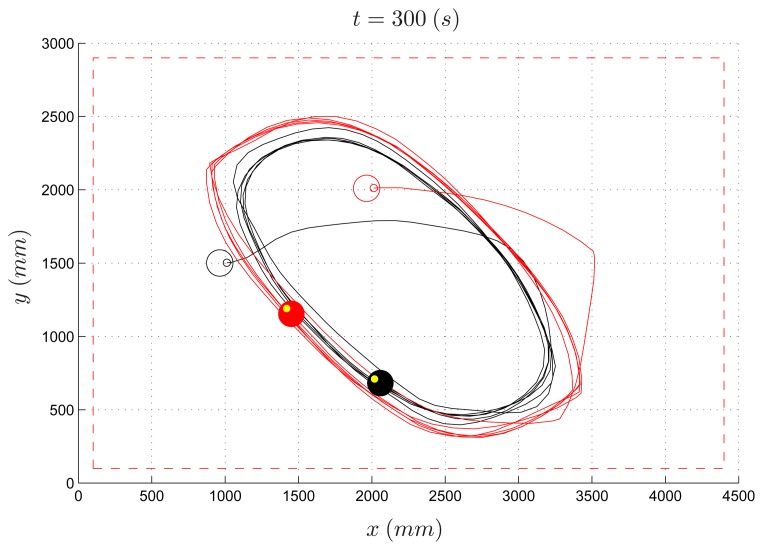
Pursuer-evader games, Algorithm 2: pursuer (black) and evader (red) with a PE speed ratio equal to 80%.

The same PE environment has been used by undergraduate students to design more sophisticated control laws for the evader (the pursuer algorithm is the same as in the previous experiment). As an example, an evader strategy based on the potential field method is reported (*Algorithm* 2). Figures 14 and 15 depict the path of two experiments where the speed ratio is set to 50% and 80%, respectively. Differently from the previous example, in both cases the evader is able to escape the pursuer.

**Figure 16.**
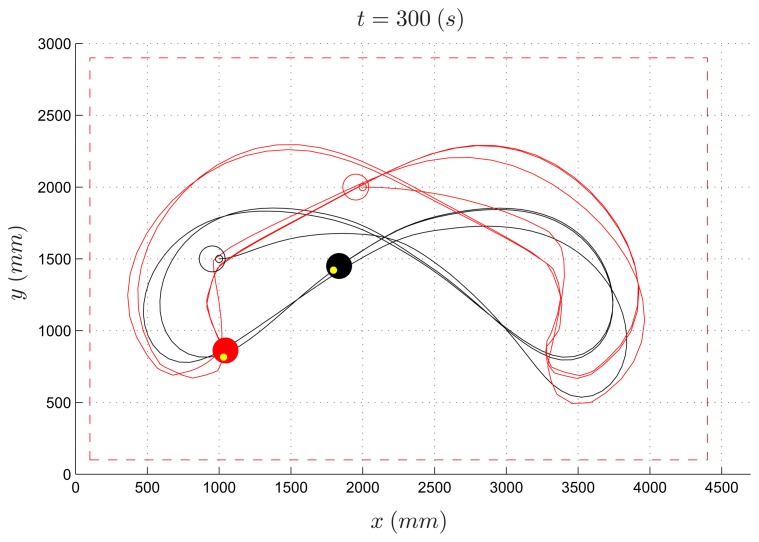
Pursuer-evader games, Algorithm 2: pursuer (black) and evader (red) with a PE speed ratio equal to 50% (simulated experiment).

In Figure 16, the simulated experiment for a speed ratio of 50% is reported. Contrary to the previous experiment, by comparing the simulated trajectory with the real one (Figure 14), one can observe that the two paths differ significantly. It is worth remarking that this is due to the complex dynamics of the overall system resulting from the coupling of the robot kinematics with the potential-based control law implemented in Algorithm 2. This is a typical situation where a virtual experiment is not able to reproduce a real one. In this case, practicing on real devices turns out to be very instructive.

### 5.2. Collision Avoidance

**Figure 17.**
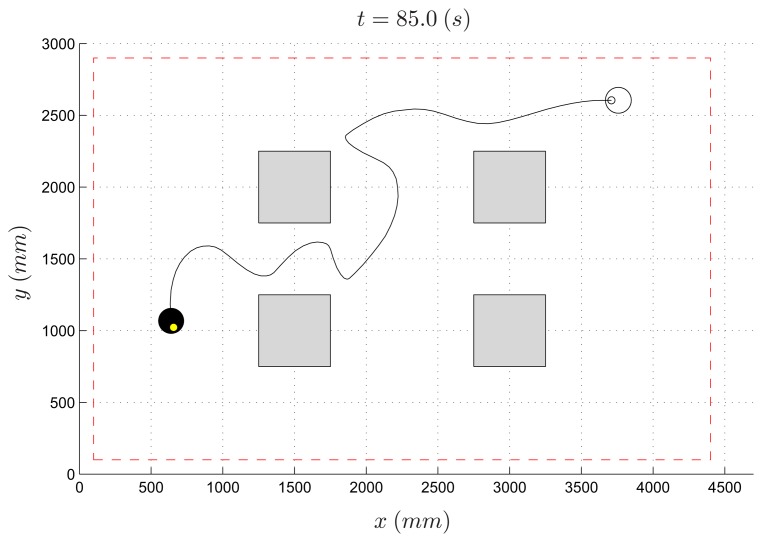
Collision avoidance: single-robot and four obstacles (shaded regions): initial robot pose (empty circle), final robot pose (filled circle), robot path (solid line).

The possibility of including virtual obstacles in the environment allows one to easily test and compare different collision avoidance algorithms. This application is specifically designed to stimulate the students to tackle the collision avoidance problem on a real testbed, without the risk of causing damages to the actual vehicles. In the example presented hereafter, an artificial potential field method, like the one described in Section 4.2, is used to compute the instantaneous desired velocity vector of robots. However, since the vehicles move according to the unicycle-like kinematics Equation (1), their velocity vector cannot be arbitrarily set due to the nonholonomic constraints. Hence, a further step is necessary to generate the references of the linear speed υ and angular speed ω, given the nominal velocity provided by the planning algorithm. To this purpose, a proportional controller has been implemented aiming to align the robot heading θ*_i_* to the angle of the desired velocity vector. Figure 17 shows the path of an experiment involving one robot and four virtual obstacles. The robot starts at position *p_i_* = [3.70 2.70]*^T^* and has to reach the target placed at *p_t_* = [0.65 1.00]*^T^*. It can be observed that in 85 s the robot succeeds in reaching the desired target while at the same time avoiding the virtual obstacles. The effects of the discrete-time implementation of the algorithm (with a sampling time *T_s_* = 1 s), as well as of the nonholonomic kinematics of the vehicle, are clearly visible in the second half of the robot path. The unnatural oscillation between the last two obstacles is due to impossibility for the robot to follow exactly the direction of fastest descent of the potential function, because of kinematic constraints and discrete-time velocity updates. The final robot position (denoted by a small light point inside the black circle in Figure 17) is slightly different from the desired one, since the task is considered accomplished when the robot is close enough to the target (in this experiment, the threshold is set to 1 cm).

This application of the mobile robot facility is suited to both introductory control courses, in which one can just ask the students to design a driving strategy preventing the robot from hitting the obstacles, and to more advanced courses, where different types of path planning techniques can be taught and tested.

### 5.3. Multi-Agent Motion Coordination

**Figure 18.**
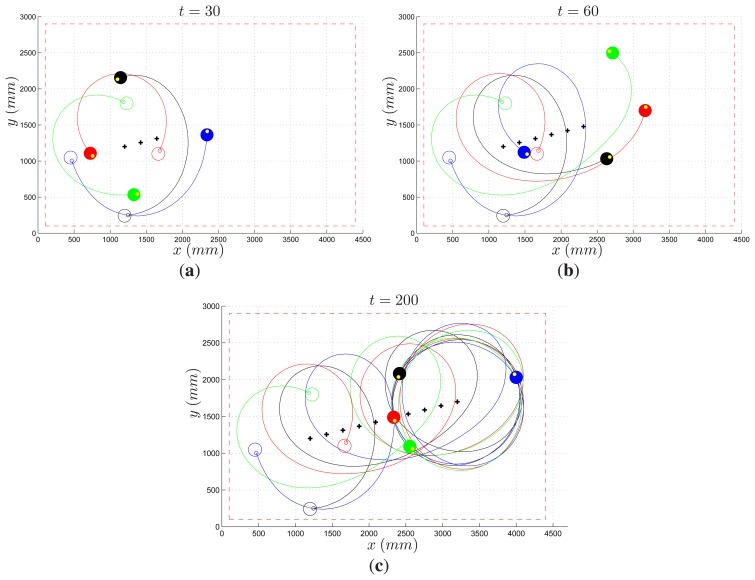
Collective circular motion: three snapshots taken at time *t* = 30 s (**a**); *t* = 60 s (**b**); and *t* = 200 s (**c**). Initial (empty circle) and current (filled circle) robot position; actual robot path (solid line); reference beacon (cross).

The first experiment concerns a decentralized control law for the collective circular motion of multi-agent systems [33]. The objective is to put the vehicle in rotation about a static or moving reference beacon, while at the same time keeping a minimum distance to the next rotating vehicle and avoiding collisions among members of the team. The problem becomes harder if one explicitly considers a number of constraints naturally arising in practice from technological limitations. It is supposed that robots are indistinguishable, and that each agent can only take range and bearing measurements with respect to “close enough” neighbors, *i.e.*, vehicles falling inside the field of view of its sensors. The remote lab provides all the necessary facilities to test such kind of control laws, allowing one to simulate sensors featuring limited field of view and indistinguishable robots. Figure 18a-c refer to an experiment of collective circular motion with the control law proposed in [33], for a team of four agents. In this case, a slowly moving reference beacon (marked with a cross) was considered. It can be seen how the vehicles proceed spiraling about the moving beacon and finally settle on a circle as the reference stops.

**Figure 19.**
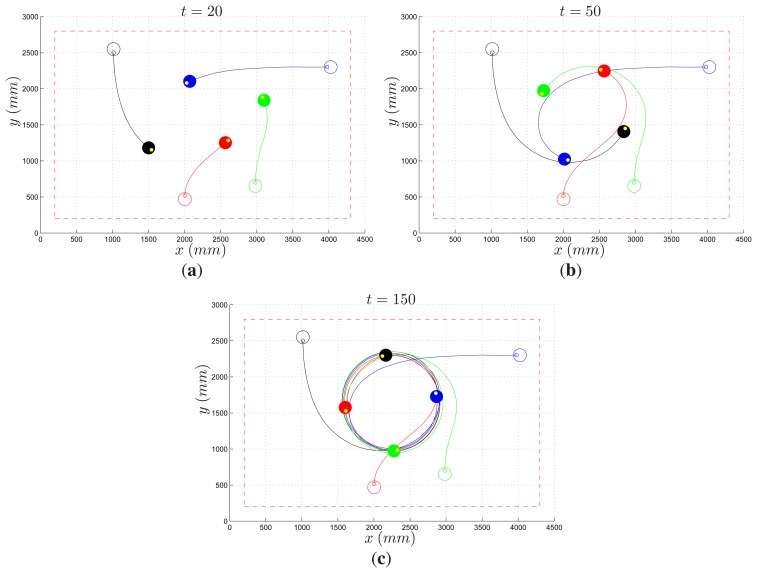
Cyclic pursuit: Three snapshots taken at time *t* = 20 s (**a**); *t* = 50 s (**b**); and *t* = 150 s (**c**). Initial (empty circle) and current (filled circle) robot position; actual robot path (solid line).

A second experiment of motion coordination concerns the cyclic pursuit problem. Differently from the previous scenario, in this case robots are distinguishable and ordered, and each agent has to pursue the next one, with the last pursuing the first one and thus closing the ring [32]. Robots are labeled from 1 to 4, and agent i has to follow agent *i* +1 (modulo 4). Depending on the control law driving each vehicle, regular formations of the platoon may occur. In the most interesting scenario, the team coordination must be achieved in a decentralized fashion, without the aid of a global controller that has access to the full state of the system. Figure 19a-c reports the results of an experiment where the cyclic pursuit algorithm studied in [32] has been implemented. Roughly speaking, the forward and angular speed of a vehicle are proportional to the distance and direction of its preceding neighbor, respectively. By properly selecting the ratio of the gains of the two controllers, it is possible to achieve a team configuration where the vehicles rotate equally spaced on a circle, like in Figure 19.

The previous examples show how the proposed setup can be effectively used to evaluate the performance of different multi-agent control strategies in a real-world scenario. This turns out to be especially useful when analyzing the effects of many uncertainty sources often neglected in the control design phase but usually occurring in practice. An additional benefit of the developed architecture is the possibility to implement the desired control strategy directly in the Matlab programming language, thus tremendously speeding up the implementation phase. As an example, the control law of the cyclic pursuit experiment took less than ten lines of Matlab code.

## 6. Conclusions

The proposed multi-robot setup, embedded in the ACT remote laboratory, is a versatile resource which has proven to be useful to both students and researchers. The proposed framework can be a powerful aid when teaching robotics courses. As a matter of fact, it allows students to perform experiments with real robots very easily. Control laws can be coded directly in the Matlab programming language, no specific low-level software/hardware knowledge is required. At the same time, researchers can immediately test new multi-agent control strategies and evaluate their effectiveness in a real-world scenario, thus bridging the gap between simulation and experimental validation.

Several new features and extensions of this setup are currently under investigation. Since some platforms are discontinuing the support of Java, different technologies for implementing the graphical interface could be considered, like, e.g., HTML5 [36]. Given the encouraging results obtained in the past, the remote lab is undergoing suitable modifications in order to organize worldwide student competitions on relevant multi-robot problems. Moreover, the addition of a user-friendly graphical interface, allowing the user to drag and drop the sensory equipment of the robots (possibly different from one another) and the obstacles present in the environment (possibly moving or partially known), will be the next steps towards the development of a fully functional augmented-reality remote lab, where heterogenous real robots equipped with virtual sensors move in real environments populated with virtual obstacles.

## Figures and Tables

**Table 1.** Physical characteristics of vehicles.

Parameter	Value
Size (L × W × H)	0.18 × 0.18 × 0.19 (m)
Weight	0.76 (kg)
Wheel distance	0.1 (m)
Wheel radius	0.017 (m)
Max linear speed	0.2 (m/s)
Max angular speed	4.0 (rad/s)
